# Wooden Toothpicks

**Published:** 1882-06

**Authors:** Kate C. Moody


### WOODEN TOOTH-PICKS.

Editor Register:—The use of the pick is acknowledged by all to be a good thing, and quite necessary to the proper cleaning of the teeth; but, like almost any other good thing, when used carelessly, may become the source of considerable suffering, as shown by the following incident.

A young man came into our office with a very sore tooth, an upper left bicuspid. On examination, it was found to be quite loose in the socket, the gum a good deal inflamed, with some pus exuding from beneath the margin; tooth very sore to the touch.

Note was taken of the fact that there was no filling or decay in that or adjoining teeth. At first salivary calculus was suspected, and a careful examination with a fine instrument, was made.

There was an entire absence of all such deposit; but at a point about half way up the root was detected something which, after a good deal of fishing, was brought to light, and proved to be a portion of a *wooden tooth-pick*, about a quarter of an inch in length, which had been broken off in picking the teeth.

After its removal, a syringe full of tepid water was thrown about the tooth, and up under the gum, and the patient dismissed. In two or three days the tooth was firm in its socket, and entirely well.

The moral of this is *not, beware of wooden tooth-picks,* but beware of *breaking them off* about the necks of the teeth.

And we might add, also, beware of a careless diagnosis, as the young man came in fully expecting to have the tooth extracted, and would have willingly submitted without any preliminary examination.

MRS. KATE C. MOODY.

